# Avian Influenza (H5N1) Outbreak among Wild Birds, Russia, 2009

**DOI:** 10.3201/eid1602.090974

**Published:** 2010-02

**Authors:** Kirill Sharshov, Nikita Silko, Ivan Sousloparov, Anna Zaykovskaya, Aleksander Shestopalov, Ilia Drozdov

**Affiliations:** State Research Center of Virology and Biotechnology, Koltsovo, Russia

**Keywords:** Avian influenza, H5N1, outbreak, wild birds, viruses, Russia, letter

**To the Editor:** Highly pathogenic avian influenza (HPAI) virus (H5N1) has been endemic in poultry in Southeast Asia since 2003 (*1*). In April 2005, an outbreak of influenza virus (H5N1) infection was detected in wild birds on Qinghai Lake in western China (*2*). Subsequently, the Qinghai-like (clade 2.2) HPAI virus (H5N1) lineage was detected in wild birds and poultry in many countries (*1*,*3*,*4*). The source of these introductions, although still debated, is likely through bird migration (*5*).

In June 2006, an influenza (H5N1) outbreak was detected in wild birds on Uvs-Nuur Lake in western Siberia, Russia. We showed that A/duck/Tuva/01/2006, isolated during the outbreak, was highly pathogenic for chickens and mice and belonged to the Qinghai-like group (2.2 clade) (*6*).

The first case of Fujian subclade 2.3.2 influenza virus (H5N1) lineage in the Russian Far East was recorded in April 2008 (*7*). Before this case, no HPAI (H5N1) outbreaks of the Fujian lineage had been reported in Russia.

In June 2009, an outbreak of HPAI in wild birds was recorded in Mongolia (*4*) and on Uvs-Nuur Lake in Russia. RNA extracted from organs (liver, spleen, intestine) of 10 dead birds belonging to 4 species (great crested grebe [*Podiceps cristatus*], little grebe [*Tachybaptus ruficollis*], black-headed gull [*Larus ridibundus*], and spoonbill [*Platalea leucorodia*]) was positive for type A influenza RNA and for the H5 subtype by real-time reverse transcription–PCR (*8*). We isolated 2 viruses from embryonated specific antibody–negative fowl eggs. Hemagglutination (HA) and neuraminidase (NA) inhibition assays with monospecific antiserum confirmed the H5N1 subtype. Viruses were designated as A/black-headed gull/Tyva/115/2009 and A/great crested grebe/Tyva/120/2009, and sequences of their HA and NA segments were defined. No HPAI virus (H5N1) was found in cloacal swabs obtained from 36 live birds (of the 4 species listed above) from Uvs-Nuur Lake.

Phylogenetic analysis (*9*) of the HA gene (Figure) showed that viruses belong to clade 2.3.2. These viruses are clearly distinguishable from the HPAI viruses previously isolated in this Russian region in 2006, A/duck/Tuva/01/2006 (clade 2.2) but are more related to A/whooper swan/Mongolia/8/2009 and A/whooper swan/Mongolia/2/2009. For the NA gene, isolated viruses were most closely related to viruses found in Mongolia. Analysis of NA protein determined that the viruses found are sensitive to NA inhibitors.

**Figure:**
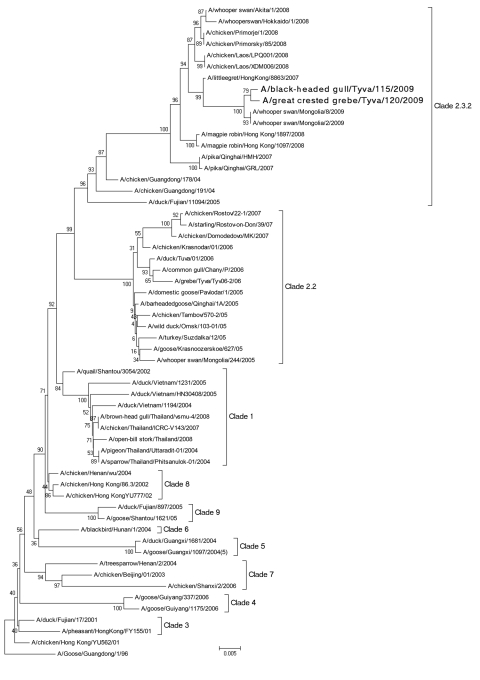
Phylogenetic tree constructed by neighbor-joining analysis (no. replications ×600) of the hemagglutinin gene segment of representative influenza virus (H5N1) isolates. Taxon names of the viruses isolated in Russia in 2006 and 2009 are in **boldface**. Scale bar indicates genetic distance.

Both viruses were shown to be highly pathogenic for chickens (intravenous pathogenicity index 3). This finding is consistent with the results of the sequence analysis of the HA gene. The HA protein possesses a series of basic amino acids (PQRERRRKR) at the cleavage site. Several amino acid changes were found between HA of investigated viruses and viruses from clade 2.3.2 that were isolated in Russia in 2008. However, the receptor-binding site of HA (positions 222–224) was not changed.

The spread of HPAI (H5N1) west across the globe has caused serious debates on the roles of migratory birds in virus circulation (*2*,*5*,*7*). In the 2009 outbreak we describe, we doubt that wild birds were infected from local poultry because domestic poultry are not present in the Uvs-Nuur Lake region and there have been no reports of HPAI among poultry in Russia since early 2008. We suggest that wild birds brought the virus to Uvs-Nuur Lake from outside the country. Because prior to June 2009 the only case of new Fujian sub-clade 2.3.2 influenza virus (H5N1) lineage was in the Russian Far East, we believe that the virus isolated in 2009 from Uvs-Nuur Lake was probably introduced by wild birds that wintered in Southeast Asia.

Many different bird species stop at Uvs-Nuur Lake during the spring and fall migrations. Qinghai-like viruses were introduced to the region from central China by wild birds in 2006 (*6*). The introduction of the H5N1 Fujian-lineage to the lake 3 years later shows further evidence that Uvs-Nuur Lake is an major area for wild bird migration and breeding and hence an environment that could potentially support the introduction of influenza virus variants from migrating wild birds. Bodies of water such as Qinghai Lake and Uvs-Nuur Lake may play a major role in the circulation of avian influenza. Therefore, we continue to study new outbreaks thoroughly and take into account the ecology and pathobiology of the species involved. Areas where large numbers of birds congregate should be closely monitored because these areas could serve as the breeding ground for avian influenza virus variants that might spread globally. Additionally, we must keep in mind that wild bird species can vary greatly in their response to HPAI and that naturally resistant waterfowl could serve as vectors for the introduction of HPAI into new locations (*1*,*2*,*5*,*7*).

Because wild birds can be involved in virus introduction, continuing surveillance is warranted. Detection of any influenza A (H5N1) virus in wild birds in a new region should be immediately followed up with efforts to characterize the virus and to control the spread of new HPAI viruses.
